# Chemotactic Bacteria Facilitate the Dispersion of Nonmotile Bacteria through Micrometer-Sized Pores in Engineered Porous Media

**DOI:** 10.1021/acs.est.2c03149

**Published:** 2022-09-14

**Authors:** María Balseiro-Romero, Ángeles Prieto-Fernández, Leslie M. Shor, Subhasis Ghoshal, Philippe C. Baveye, José Julio Ortega-Calvo

**Affiliations:** † Instituto de Recursos Naturales y Agrobiología de Sevilla (IRNAS), Consejo Superior de Investigaciones Científicas (CSIC), Avda. Reina Mercedes 10, 41012 Sevilla, Spain; ‡ Instituto de Investigaciones Agrobiológicas de Galicia (IIAG), Consejo Superior de Investigaciones Científicas (CSIC), Avda. de Vigo s/n, 15705 Santiago de Compostela, Spain; § Department of Chemical and Biomolecular Engineering, University of Connecticut, Castleman Building Rm. 224, Connecticut 06269-3237 Storrs, United States; ∥ Department of Civil Engineering, McGill University, 817 Sherbrooke Street West, Montreal, Quebec H3A 0C3, Canada; ⊥ Saint Loup Research Institute, 79600 Saint Loup Lamairé, France

**Keywords:** microbe−microbe cotransport, chemotaxis, bioaccessibility, micrometer-sized pores, hitchhiking

## Abstract

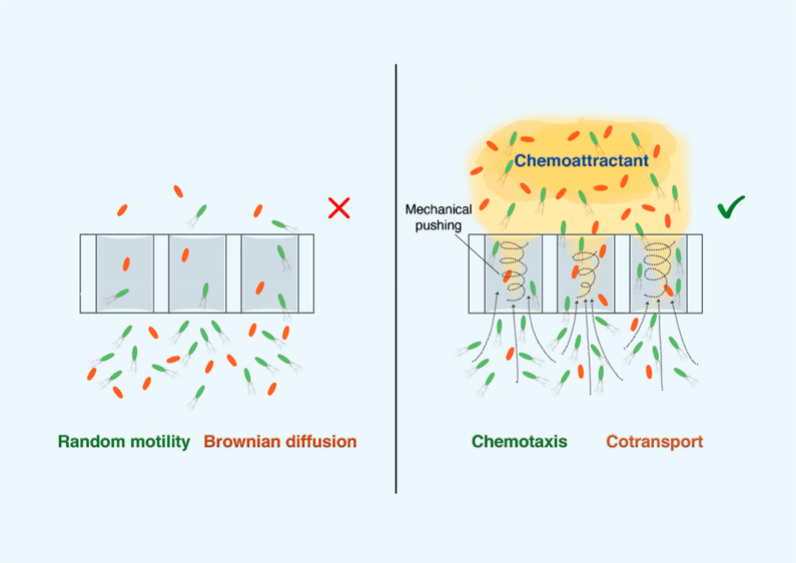

Recent research has demonstrated that chemotactic bacteria can disperse inside microsized pores while traveling toward favorable conditions. Microbe–microbe cotransport might enable nonmotile bacteria to be carried with motile partners to enhance their dispersion and reduce their deposition in porous systems. The aim of this study was to demonstrate the enhancement in the dispersion of nonmotile bacteria (*Mycobacterium gilvum* VM552, a polycyclic aromatic hydrocarbon-degrader, and *Sphingobium* sp. D4, a hexachlorocyclohexane-degrader, through micrometer-sized pores near the exclusion-cell-size limit, in the presence of motile *Pseudomonas putida* G7 cells. For this purpose, we used bioreactors equipped with two chambers that were separated with membrane filters with 3, 5, and 12 μm pore sizes and capillary polydimethylsiloxane (PDMS) microarrays (20 μm × 35 μm × 2.2 mm). The cotransport of nonmotile bacteria occurred exclusively in the presence of a chemoattractant concentration gradient, and therefore, a directed flow of motile cells. This cotransport was more intense in the presence of larger pores (12 μm) and strong chemoeffectors (γ-aminobutyric acid). The mechanism that governed cotransport at the cell scale involved mechanical pushing and hydrodynamic interactions. Chemotaxis-mediated cotransport of bacterial degraders and its implications in pore accessibility opens new avenues for the enhancement of bacterial dispersion in porous media and the biodegradation of heterogeneously contaminated scenarios.

## Abstract 2

The chemotaxis-mediated cotransport of nonmotile cells through cell-size restricted micropores was demonstrated.

## Introduction

Until the early 1990s, bacteria were generally considered to be adsorbed and incapable of moving significantly on their own in natural and engineered porous media, including soils.^1^ Breakthrough experiments in porous columns under saturated^2,3^ or unsaturated^4^ conditions indicated that bacteria could be transported along passively within the percolating liquid phase or even attached to the skin of earthworms and move in soils along with them.^5^

Perceptions in this respect have changed drastically in the last two decades. Experimental evidence suggested that bacteria could attach not just episodically to earthworms but also to fungal hyphae, and move along with them in different porous media.^6−8^ In addition, evidence of bacteria able to slide^9^ or glide^10^ on solid surfaces in soils and other porous media suggested that these organisms might be far more mobile than anticipated, although these types of motion, like the traditional Brownian movement, are not particularly effective for long-range dispersion. Flagellated bacteria^11,12^ respond to gradients of diverse stimuli through tactic responses, including chemical concentration gradients, and they can actively disperse throughout water (swimming) and on soil–water interfaces (swarming) toward sources of nutrients. In recent years, through the development of novel experimental techniques based on the thin sectioning of resin-impregnated soil samples combined with X-ray computed tomography,^13,14^ it was confirmed that bacterial cells are indeed able to move appreciably and surprisingly fast on their own through the pore space.^15^

Until recently, the dispersion of bacteria in soils and other natural porous media was thought to be restricted to relatively large pore sizes,^16^ significantly larger than the typically micrometric size of bacterial cells, where bacteria can freely swim. This restriction was one of the key factors that explained the recalcitrance of stable organic matter in soils^17^ residing in small pores. Similarly, in polluted porous media, the low dispersion and high deposition rates of bacteria in small pores was advocated as one of the key reasons for the limited bioavailability of organic xenobiotics for a long time.^11^ However, it has been recently demonstrated that the use of suitable chemoeffectors can decrease bacterial deposition rates in porous media^18^ and that motile bacterial cells are able to penetrate micrometer-sized pores in the presence of chemical effector gradients.^19^

In parallel with this change in perceptions, it has been demonstrated that some nonmotile microbes have the ability to accompany motile microbes.^20−22^ However, the exact mechanisms of this “hitchhiking” or microbe–microbe cotransport are still largely unclear, although some mechanisms have been identified, including mechanical pushing, direct surface attachment to motile cells, direct attachment to flagella, and cell internal transport.^23,24^ The “cargo–carrier” association can be beneficial for both parties. Specifically, nonmotile microbes may spread to otherwise inaccessible nutrients or carbon sources, and both may be positively influenced by the coupling of their metabolic capabilities, communal sharing of excreted molecules, or community antibiotic resistance enhancement, resulting in the improvement of growth and fitness of the partners.^21,25^ A context in which cotransport may be significant relates to the bioavailability of organic contaminants in soils and sediments and consequently to pollutant biodegradation and soil remediation attainment. Enhancing the dispersion of pollutant-degrading bacteria via cotransport with directionally moving microbes is an attractive and novel strategy to be applied in bioremediation of soils contaminated with hydrophobic organic contaminants (HOC). An improved microbial dispersion will enhance the access of a variety of degrading microbes (motile or not) to distant pollutant sources and relatively nonbioaccessible pores. Maximizing and optimizing the motility and bacterial tactic response utilizing suitable chemical effectors (chemoeffectors) appears essential. Chemoeffectors are known to reduce the bacterial depositions rates in porous media. Random cell swimming, which is typically characterized by short paths and spontaneous changes in the directions of swimming, tends to be smoothed in the presence of chemoeffectors and favors long-distance bacterial dispersion within porous environments.^18,26−28^ Chemoeffectors positively impact the chemotactic response of carrier cells, and they will trigger bacterial transport and, consequently, hitchhiking.

In this general context, the objective of the research reported on in this article was to investigate the chemotaxis-mediated cotransport of nonmotile cells of HOC-degrading bacteria (*Mycobacterium gilvum* VM552 and *Sphingobium* sp. D4) in micrometer-sized pores, and *Pseudomonas putida* G7 cells, a motile and naphthalene-degrading strain, was used as a carrier. This objective was particularly examined using devices that mimicked the restriction in cell transport in porous media, including bioreactors, composed of two chambers that were separated by membranes with 3, 5, and 12 μm pore sizes, and polydimethylsiloxane (PDMS) microarrays (20 μm wide × 35 μm high × 2.2 mm long). Sodium salicylate, a naphthalene degradation metabolite, and γ-aminobutyric acid, a common component of root exudates of sunflower (a plant species that was previously utilized in polycyclic aromatic hydrocarbon (PAH) bioremediation procedures together with *P. putida* G7),^29^ were used as chemoeffectors. These compounds were previously demonstrated to trigger the chemotactic response of *P. putida* G7 in the direction of a positive concentration gradient.^18,19^ To our knowledge, this is the first study that investigated the microbe–microbe cotransport of nonmotile cells that exhibit HOC-degrading capacities through micrometer-sized pores and the enhancement of this transport due to the directional movement of a chemotactic bacteria (which also exhibits degrading capacities). This study will have greater implications on bacterial pore accessibility and on biodegradation enhancement. The design of inoculants of bioremediation strategies should prioritize not only those with degradation capacities but also those which may actively disperse (or be cotransported).

## Materials and Methods

### Chemical Reagents

Sodium salicylate (SAL; PanReac AppliChem, Spain) and γ-aminobutyric acid (GABA; Merck KGaA, Germany) were used as chemoeffectors to induce the tactic response and the directional movement of *P. putida* G7 cells.

### Bacterial Strains and Cultivation Media

The hexachlorocyclohexane (HCH)-degrader strain *Sphingobium* sp. D4 was isolated from an HCH-contaminated soil (Porriño, Spain).^30,31^ The multiple PAH-degrader *Mycobacterium gilvum* VM552 and the chemotactic naphthalene-degrader *Pseudomonas putida* G7 were obtained from the permanent collection at the Instituto de Recursos Naturales y Agrobiología de Sevilla (Spain). *Sphingobium* sp. D4 was cultured in 869 media^32^ (1:5 diluted, *v*/*v*) from glycerol primary stock, and *M. gilvum* VM552 and *P. putida* G7 were grown in tryptic soy broth (PanReac AppliChem, Spain) from bacterial biomass that was maintained on tryptic soy agar plates (PanReac AppliChem, Spain) and minimal salt medium (MSB)^33^ agar plates in the presence of naphthalene crystals as a source of carbon, respectively. The strains were incubated at 30 °C and 150 rpm on a rotary shaker for 24 h in the case of *Sphingobium* sp. D4 and overnight (∼12 h) in the cases of *M. gilvum* VM552 and *P. putida* G7 (which was collected in the early stationary phase when stable generalized motility was achieved). The stability of *P. putida* G7 motility was checked microscopically throughout the experiments.

### Cell Size and Surface Properties

The length and breadth of the cells used for the experiments were determined in microscope images obtained using a camera Axiocam 305 color (Zeiss, Germany; interface, Zen software, blue edition) that was connected to a phase-contrast inverted microscope AxioVert.A1 (Zeiss, Germany). Cell surface hydrophobicity was calculated according to the bacterial adhesion to hydrocarbons (BATH) method.^34^ Briefly, 4 mL of the bacterial suspension (optical density at 600 nm (OD_600nm_) = 0.1–0.2; 10^7^ cells mL^–1^) was vortex-mixed with 1 mL of hexadecane for 2 min. The phases were left to separate for at least 15 min, and the cell density in the aqueous phase was determined by OD_600nm_ and/or Neubauer chamber counting. Cell hydrophobicity was calculated as the percentage of initial cells partitioned to hexadecane. A Zetasizer Nano ZSP analyzer (Malvern, UK) was used to determine the zeta potential of the bacterial suspensions based on the electrophoretic mobility by laser Doppler microelectrophoresis.

### Cell Staining and Quantification of Cell Density Using Fluorescence Spectroscopy

A faster quantification of the nonmotile cell concentrations was obtained with this specifically developed method, which will be described further in detail, compared with viable colony forming units (CFU) plate counting or Neubauer chamber counting. This method for cell density quantification by fluorescence spectroscopy was more specific and precise when colony differentiation in plates and/or cell differentiation under microscope observations was challenging and tended to be subjective for mixed suspensions with *P. putida* G7. *M. gilvum* VM552 and *Sphingobium* sp. D4 cells were stained with acridine orange (AO; Merck KGaA, Germany) following the method described by Guo et al.^35^ Briefly, *M. gilvum* VM552 and *Sphingobium* sp. D4 cells (cultured as previously described) were collected by centrifugation (8000 and 6000 rpm, 10 min, respectively), washed once with MSB medium, and resuspended to OD_600nm_ = 0.5. Then, 9 mL aliquots of the cultures were vortex-mixed with 1 mL of a 0.02% (*w*/*v*) filtered-sterilized AO solution for 3 min. These suspensions were left in the dark for 15 min, and then, 3 subsequent washing cycles were performed to eliminate free stain residues: centrifuging M. *gilvum* VM552 and *Sphingobium* sp. D4-stained cells (8000 and 6000 rpm, 5 min, respectively), discarding the supernatant, adding 9.7 mL of MSB, and vortex-mixing the sample to resuspend the biomass.

Fast quantification of the stained cell concentration was carried out using a fluorescence spectrometer (F-2500, Hitachi, Japan), in which the emission spectra for AO were collected from 400 to 700 nm, 473 nm was used as the excitation wavelength, and 2.5 and 20.0 nm excitation and emission slits were used, respectively. Due to cell suspension turbidity, the fluorescence of AO bound to cells presented a nonlinear response to concentration (i.e., inner filter effect, IFE).^36^ IFE-corrected fluorescence was calculated by multiplying the observed fluorescence to a correction factor (*CF*). This factor was estimated from the linear representation of the OD_600nm_ of several *P. putida* G7 + *M. gilvum* VM552 or *Sphingobium* sp. D4 suspensions versus the ratio of the observed and the theoretical (in the absence of *P. putida* G7 cells) fluorescence intensity of *M. gilvum* VM552 or *Sphingobium* sp. D4 stained cells as follows (eqs 1 and 2, respectively; Figure S1):
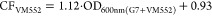
1
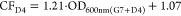
2where CF_VM552_ and CF_D4_ are the estimated correction factors for *M. gilvum* VM552 and *Sphingobium* sp. D4 fluorescence, respectively, in mixed suspensions.

### Cell Tracking and Analysis of Swimming Motility and Diffusion

The swimming motility of *P. putida* G7 cells was characterized using individual (control), and mixed suspensions of *P. putida* G7 + *M. gilvum* VM552 or *Sphingobium* sp. D4 1:1 (carrier/cargo cell ratio). Bacterial cultures, which were obtained as described above, were centrifuged, washed once with phosphate buffer (6.1 mM K_2_HPO_4_, 3.9 mM KH_2_PO_4_, and 20 μM EDTA) and suspended to a final optical density of OD_600nm_ = 0.1 of each strain. LabTrack software (BIORAS, Denmark) was used to analyze the bacterial trajectories with short videos (<5 s; 35 frames per second; pixel size, 0.173 μm × 0.173 μm) that were recorded using a camera Axiocam 305 color (Zeiss, Germany), connected to a phase-contrast inverted microscope AxioVert.A1 (Zeiss, Germany). This software identifies each cell as an individual particle and tracks its trajectory over time. This software determines individual cell parameters such as the instantaneous speed (the speed that each individual cell has at each video frame; μm s^–1^) and position of cells in each frame (coordinate *X* and *Y*; μm). For each scenario, 20 representative cell trajectories were selected. *P. putida* G7 cells, with a mean instantaneous speed lower than 17 μm s^–1^ were discarded.^18^ This boundary speed allowed those immotile cells to be discarded according to the video resolution (i.e., cells that moved less than 2–3 pixels among video frames). Although *M. gilvum* VM552 or *Sphingobium* sp. D4 are nonmotile under the scenarios tested (only Brownian diffusion was observed), the apparent motion and displacement driven by the interactions with *P. putida* G7 was also analyzed in bulk suspensions, as previously described. For this, videos of bacterial suspensions were recorded under fluorescent light to easily discriminate the stained *M. gilvum* VM552 or *Sphingobium* sp. D4 cells from *P. putida* G7 cells. *P. putida* G7-free controls were also analyzed to characterize the Brownian movement of the nonmotile cells.

From the LabTrack raw data of individual cells, the average population values of the following parameters were calculated to characterize bacterial trajectories: a) the mean instantaneous swimming speed observed for individual cells during the video duration (and the maximum found among selected individuals; *n* = 20); b) the net displacement in *X* and *Y* coordinates per time, which was calculated as the difference between the maximum and minimum values found during the cell trajectory (and the maximum value among individuals); and c) the frequency of acceleration events, which corresponds to the number of swimming speed peaks that were >60 μm s^–1^ for *P. putida* G7 and >20 μm s^–1^ for *M. gilvum* VM552 and *Sphingobium* sp. D4, during the trajectory duration (and the maximum value among individuals).

The chemical-in-capillary (CC) method was used to characterize the chemotactic responses of *P. putida* G7 cells toward chemoeffectors (SAL or GABA, 10 mM) as described elsewhere,^18^ and the bacterial suspensions were the same as those for motility characterization (in triplicate). The chemoeffector concentration gradient that was generated at the tip of the capillaries triggered the tactic response of *P. putida* G7, and viable cells that entered the capillaries after 30 min of incubation were quantified as CFU mL^–1^ plated on tryptic soy agar plates. The tactic factor was calculated as the ratio between the CFU mL^–1^ inside the capillaries in the presence of chemoeffectors and the corresponding metric in chemoeffector-free controls.

### Microbe–Microbe Cotransport Experiments in Micrometer-Sized Scenarios

**Figure 1:**
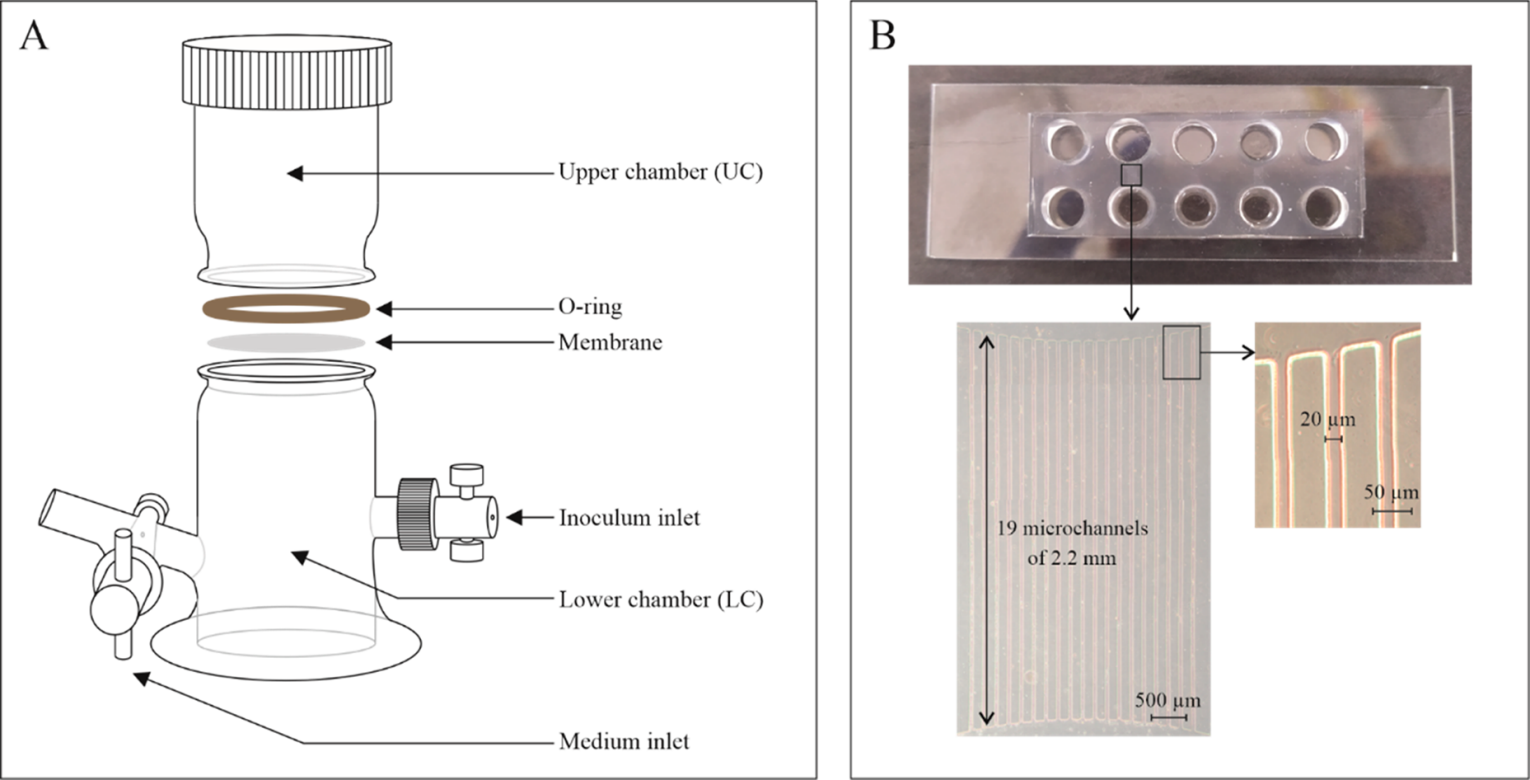
Schematic representation of (A) the bioreactors and (B) the capillary microarrays used for microbe–microbe cotransport experiments.

Several experiments were carried out to demonstrate the cotransport of *M. gilvum* VM552 and *Sphingobium* sp. D4 within a front of *P. putida* G7 motile through micrometer-sized pores. A chemoeffector chemical gradient was created in those scenarios to trigger the chemotactic response of *P. putida* G7, and to create a continuous and directional flow of motile cells. Several devices (i.e., bioreactors and PDMS capillary microarrays) were used to mimic the microporous systems in soil environments. The devices and the corresponding experimental setups are described in further detail below and in Figure 1.

Bioreactors, as described elsewhere,^37,19^ consist of two glass-chambers that are separated by 9 μm thick polycarbonate membrane filters with different micrometer-sized pores (i.e., 3, 5, and 12 μm): the lower chamber, in which cell suspensions were injected at a certain optical density, and the upper chamber, in which chemoeffectors (SAL or GABA at 10 mM) were added and transported cell quantification was performed (Figure 1A). The addition of the chemoeffectors to the upper chamber created a concentration gradient through the membranes, and this gradient triggered the chemotactic motility of *P. putida* G7. The vertical configuration of the chambers prevented the transport of *P. putida* G7 by free convection. The bioreactors were filled with 18 mL of MSB medium, and this corresponded to 3 and 15 mL in the upper and lower chambers, respectively, including 1 mL of bacterial suspensions (grown and washed as previously described), which were injected in the lower chamber as follows: individual *P. putida* G7, *M. gilvum* VM552, or *Sphingobium* sp. D4 (controls) and mixed suspensions at carrier/cargo cell ratio of 1:1 (OD_600nm_ = 0.1 for both strains) or 10:1 (OD_600nm_ = 0.3 for *P. putida* G7 and OD_600nm_ = 0.03 for *M. gilvum* VM552 or *Sphingobium* sp. D4). Immediately after the cells were injected, the chemoeffector (SAL or GABA) was introduced in the upper chamber at 10 mM. Controls with no chemoeffector were also set up to check bacterial transport due to the dispersion and random motility of *P. putida* G7. The bioreactors were incubated under static conditions for 8 h at room temperature (22 ± 2 °C) in the dark (to avoid AO photodegradation). Every hour, the cell concentration in the upper chamber (which was previously gently homogenized by pipetting strokes) was quantified through Neubauer chamber cell counting (quantification of *M. gilvum* VM552 or *Sphingobium* sp. D4 nonmotile cells, under fluorescent light), OD_600nm_ (as total cell density), and fluorescence spectroscopy measures (fast quantification of *M. gilvum* VM552 or *Sphingobium* sp. D4 cell concentration, as previously described). The transport rate of *M. gilvum* VM552 or *Sphingobium* sp. D4 at each sampling time was calculated as the concentration of nonmotile cells in the upper chamber (*C*; CFU mL^–1^) divided by the total concentration of those cells in the whole bioreactor (*C*_0_). *C*_0_ was calculated from the number of cells added in the lower chamber at the beginning of the experiment and the total volume of the liquid in the bioreactor. Experiments were performed at least in duplicate.

The diffusion coefficients of bacterial cells were calculated for each scenario with the method described for a Stokes diaphragm cell^38^ as follows (eq 3):
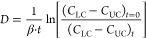
3where *D* is the diffusion coefficient of the cells (cm^2^ s^–1^), β is a diaphragm-cell constant (cm^–2^), *t* is the time (s), and *C*_LC_ and *C*_UC_ are the concentrations of cells in the lower and upper chambers of the bioreactors, respectively (cells cm^–3^). The average *D* value was calculated from the slope of the curve resulting from representing  versus the incubation time.

The constant β can be calculated for each membrane using eq 4 as follows:
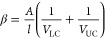
4where *A* is the area available for diffusion (cm^2^), *l* is the effective thickness of the membrane (i.e., 0.0009 cm), and *V*_LC_ and *V*_UC_ are the volumes of the lower and upper chambers, respectively (i.e., 15 and 3 cm^3^). The area available for diffusion corresponds to the area of the membrane that is occupied by pores, which was previously calculated^19^ as 0.46, 0.37, and 0.3 cm^2^, for the 3, 5, and 12 μm membranes, respectively.

The adhesion of *M. gilvum* VM552 and *Sphingobium* sp. D4 cells to membranes either in the absence or the presence of *P. putida* G7 was evaluated under similar conditions to those occurring in the bioreactors. Membranes were immersed in cell suspensions (that were prepared as for bioreactor experiments) and statically incubated for 8 h. In the case of *Sphingobium* sp. D4 in 12 μm membranes, a slight decrease in OD_600nm_ (3.9 ± 2.5%) was observed only in the presence of *P. putida* G7. Adhesion to membranes was negligible for *M. gilvum* VM552 cells in the presence and in the absence of *P. putida* G7. Adhesion of *P. putida* G7 cells was previously determined to be also negligible.^19^ Therefore, cell adhesion to membranes was not considered in cell diffusion calculations.

Furthermore, nonmotile cell growth was minimized under the experimental conditions (limiting the incubation time to 8 h). Viability controls also showed that compared to nonstained cells, the stained cells were less viable. In AO-stained suspensions, the viability of *M. gilvum* VM552 and *Sphingobium* sp. D4 cells were 2 and 1 orders of magnitude fewer respectively, compared to that of the nonstained suspensions.

Capillary arrays are PDMS-on-glass microfluidic devices comprised of bundles with 19 parallel microchannels (20 μm wide × 35 μm high × 2.2 mm long) connected to two inlet/outlet wells (Figure 1B). The arrays were produced using photolithography and soft lithography as described elsewhere.^39^ First, the microchannels were saturated with MSB media using a 5 mL glass syringe (Hamilton, USA), in which the threaded tip fit the diameter of the inner well. Excess MSB in the inlet and outlet wells was removed, and immediately filled with 100 μL of concentrated chemoeffector (GABA 100 mM, in the outlet well) and with bacterial suspensions (in the inlet well). The bacterial suspensions tested were individual *P. putida* G7, *M. gilvum* VM552, or *Sphingobium* sp. D4 (controls), and *P. putida* G7 + *M. gilvum* VM552 or *Sphingobium* sp. D4 mixed suspensions with a 1:1 carrier/cargo cell ratio (OD_600nm_ = 0.5 for all strains). Experiments were performed in duplicate. The devices were observed with an inverted microscope, and videos of the bacterial movement were recorded, as described above. When the *P. putida* G7 front reached to the outlet well and a homogeneous bacterial density was observed inside the microchannels (approximately, after 2 h), a 10 μL aliquot of the outlet suspension was observed under the microscope and was used for cell counting in a Neubauer chamber. *Sphingobium* sp. D4 AO-stained cells were used to facilitate their visual differentiation from *P. putida* G7 cells under fluorescent light.

### Statistical Analysis

SPSS Statistics (version 27; IBM, USA) was used to analyze the data. Student’s *t*-tests were performed to compare the cell tracking data obtained for *P. putida* G7, and the tests were performed individually and in the presence of nonmotile cells of *M. gilvum* VM552 and *Sphingobium* sp. D4, as well as for *M. gilvum* VM552 and *Sphingobium* sp. D4 cells, both individually and in the presence of *P. putida* G7. A multivariate ANOVA test was performed to determine the influence of different factors (pore size, chemoeffector, and *P. putida* G7 cell proportion) on the *C*/*C*_0_ of *M. gilvum* VM552 and *Sphingobium* sp. D4. Levene’s test was previously applied to check the assumption of variance equality.

## Results

### Physicochemical Surface Properties of the Bacteria

Measurements of cell sizes and the physicochemical surface properties of the bacteria, including hydrophobicity (using the BATH method) and zeta potentials (ζ) indicate (see Table 1) that all strains were negatively charged, but *Sphingobium* sp. D4 exhibits a significantly lower zeta potential compared with than that of *P. putida* G7 and *M. gilvum* VM552. *Sphingobium* sp. D4 is highly hydrophobic. In contrast, *M. gilvum* VM552 exhibits a moderate hydrophobicity, and *P. putida* G7 is hydrophilic.

**Table 1:** Cell Size and Physicochemical Surface Properties of the Bacteria Used in This Study^a^

strain	length (μm)^b^	breadth (μm)^b^	*L*/*B* ratio	ζ (mV)^c^	hydrophobicity (%)^d^
*P. putida* G7	4.22 ± 0.57	1.13 ± 0.22	3.94 ± 1.27	–35.9 ± 2.6	4.5 ± 2.1%
*M. gilvum* VM552	3.91 ± 0.99	2.22 ± 2.05	1.70 ± 1.79	–41.9 ± 3.0	51.4 ± 11.3%
*Sphingobium* sp. D4	3.49 ± 1.20	1.42 ± 0.31	2.43 ± 0.58	–8.0 ± 0.4	88.0 ± 2.0%

a The results are presented as the mean ± the standard deviation.

b *n* = 10.

c Zeta potential (*n* = 3).

d Percentage of cells partitioned to hexadecane from water suspensions (BATH method; *n* = 3).

### Quantification of the Cotransport of *M. gilvum* VM552 and *Sphingobium* sp. D4 with the Motile Cells of *P. putida* G7

**Figure 2:**
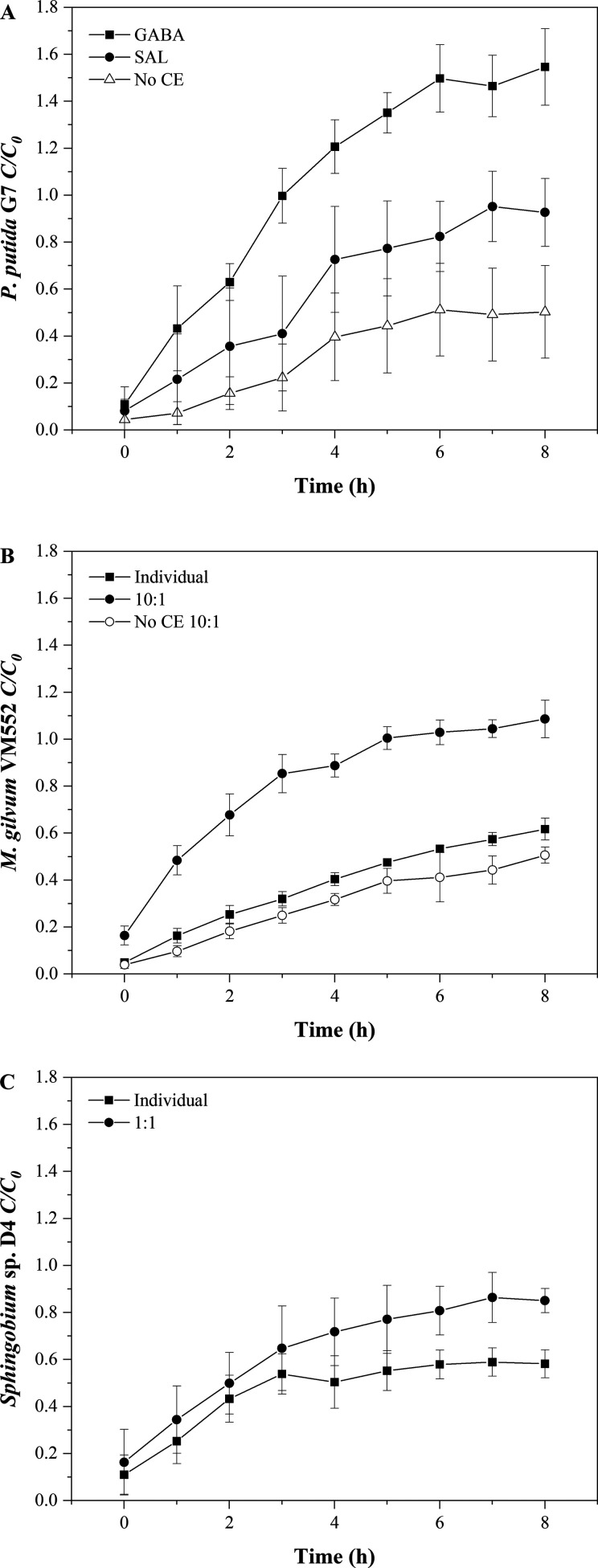
Bioreactor results of *C*/*C*_0_ of (A) *Pseudomonas putida* G7 in the presence and the absence of chemoeffector (CE), (B) *Mycobacterium gilvum* VM552, and (C) *Sphingobium* sp. D4 individually and in the presence of *P. putida* G7 (10:1 and 1:1 carrier/cargo cell ratios respectively) in the presence of 10 mM GABA in the upper chamber of 12 μm bioreactors. The results are presented as the mean ± the standard deviation.

The chemotactic response of *P. putida* G7 to chemoeffectors in the absence of nonmotile cells was determined in bioreactors (Figure 2A). Calculations of *C*/*C*_0_ and *D* are shown in Table 2. The results show a clear increase in *C*/*C*_0_ values in the presence of SAL and GABA, compared to that of the chemoeffector-free controls. In the 5 μm bioreactors, those values are slightly lower than those in the 12 μm bioreactors. As a result of the enhanced *C*/*C*_0_ values, the effective diffusivity of *P. putida* G7 cells increased an order of magnitude in the presence of chemoeffectors, compared to the value that was attributed to random walk (Table 2). The *D* values with GABA are slightly higher than those with SAL for a given pore size, and this provides evidence for the different chemotactic reaction strengths that were observed in the capillary assays (Table S1).

**Table 2:** Effect of Chemoeffectors (CE) on the Dispersion of *Pseudomonas putida* G7 Motile Cells in 5 and 12 μm Bioreactors^a^

chemoeffector	pore size (μm)	*C*/*C*_0:*t*=8h_^b^	*D* (10^–7^; cm^2^ s^–1^)^c^
no CE	5	0.23 ± 0.11	0.82
	12	0.50 ± 0.20	2.78
GABA	5	1.10 ± 0.12 (4 h)	11.22
	12	1.55 ± 0.16 (3 h)	14.81
SAL	5	0.93 ± 0.12 (5 h)	6.9
	12	0.93 ± 0.14 (5 h)	11.79

a The experimental results are presented as the mean ± the standard deviation.

b *C*/*C*_0_ after 8 h of incubation. In brackets, the time at which *C*/*C*_0_ > 0.75, if observed

c Average diffusion coefficients of P. putida G7 cells found for each scenario during the incubation time (eq 3).

Figure 2B,C illustrates the cotransport of *M. gilvum* VM552 and *Sphingobium* sp. D4 in the best selected scenarios of those detailed in Table 3. The transfer of *M. gilvum* VM552 and *Sphingobium* sp. D4 cells to the upper chamber of the bioreactor was significantly enhanced when motile *P. putida* G7 cells were present, and this occurred in the direction of the positive chemoattractant concentration gradient that was generated through the membranes. Indeed, chemoeffector-free controls were performed to demonstrate that this enhanced transport of nonmotile cells was a consequence of the *P. putida* G7 chemotactic response. As an example, Figure 2B shows that no mobilization of *M. gilvum* VM552 cells through 12 μm membranes was observed in the presence of *P. putida* G7 without any chemoeffector.

**Table 3:** Cotransport of *Mycobacterium gilvum* VM552 and *Sphingobium* sp. D4 in the Presence of *Pseudomonas putida* G7 Motile Cells after 8 h of Incubation (*C*/*C*_0:*t*=8_) in Bioreactors^a^

			VM552/D4 individual		G7 + VM552/D4 1:1		G7 + VM552/D4 10:1	
nonmotile cell	chemoeffector	pore size (μm)	*C*/*C*_0:*t*=8h_^b^	*D* (10^–7^; cm^2^ s^–1^)^c^	*C*/*C*_0:*t*=8h_^b^	*D* (10^–7^; cm^2^ s^–1^)^c^	*C*/*C*_0:*t*=8h_^b^	*D* (10^–7^; cm^2^ s^–1^)^c^
*M. gilvum* VM552	no CE	12					0.51 ± 0.03	5.32
	GABA	3	0.68 ± 0.18	4.65	0.54 ± 0.19	6.78	0.61 ± 0.15	5.19
		5	0.59 ± 0.20	4.61	0.59 ± 0.21	5.47	0.84 ± 0.06 (7 h)	7.68
		12	0.62 ± 0.05	8.70	0.82 ± 0.01 (7 h)	12.64	1.09 ± 0.08 (3 h)	36.17
*Sphingobium* sp. D4	GABA	3	0.54 ± 0.17	2.22	0.48 ± 0.16	4.25	0.68 ± 0.39	5.08
		5	0.32 ± 0.07	2.27	0.58 ± 0.05	4.27	0.43 ± 0.04	3.28
		12	0.57 ± 0.07	6.33	0.85 ± 0.05 (5 h)	18.84	0.98 ± 0.17 (5 h)	15.89
	SAL	3	0.10 ± 0.01	0.36	0.15 ± 0.01	0.69		
		5	0.19 ± 0.09	1.66	0.36 ± 0.08	2.25	0.41 ± 0.04	7.78
		12	0.19 ± 0.02	1.54	0.45 ± 0.12	3.92		

a The experimental results are presented as the mean ± the standard deviation.

b *C*/*C*_0_ after 8 h of incubation. In brackets, the time at which *C*/*C*_0_ > 0.75, if observed.

c Average diffusion coefficients of nonmotile cells found for each scenario during the incubation time (eq 3).

Generally, the significance of cotransport increased as the pore size increased (*F* = 9.691 and 2.244 for *M. gilvum* VM552 and *Sphingobium* sp. D4, respectively; *p* < 0.05) and when in the presence of stronger chemoattractants (GABA) (*F* = 7.669 for *Sphingobium* sp. D4; *p* < 0.05). The proportion of *P. putida* G7 in the mixed suspensions was only significant for *M. gilvum* VM552 (*F* = 5.517; *p* < 0.05) (Table S2). In the presence of GABA, the cotransport of nonmotile cells was very significant, but it depended on the mobilized strain. Transport of *M. gilvum* VM552 with *P. putida* G7 was much more effective than that of *Sphingobium* sp. D4: The concentration of *M. gilvum* VM552 in bioreactors was completely homogenized at the end of the experiment and eventually reached *C*/*C*_0_ > 0.75 at shortest times (e.g., 7 and 3 h incubations in 5 and 12 μm bioreactors, respectively, with a cell ratio of 10:1, Table 3). After 8 h of incubation, the concentration of *Sphingobium* sp. D4 in the upper chamber of the 12 μm bioreactors was almost completely homogenized at both sides of the membrane (*C*/*C*_0_ > 0.75 after 5 h), while it barely reached 0.6 in the 3 and 5 μm bioreactors. Accordingly, the diffusion coefficients of nonmotile cells are an order of magnitude higher in the presence of *P. putida* G7 cells and are particularly higher in the most favorable scenarios for chemotaxis (12 μm and GABA).

### Motion Analysis and the Tactic Response of Cells in Individual and Mixed Bulk Suspensions

In bulk suspensions, the motility behavior and the chemotactic response of *P. putida* G7 were not significantly modified in the presence of *M. gilvum* VM552 or *Sphingobium* sp. D4 nonmotile cells. The instantaneous cell speed (Table 4) of *P. putida* G7 and its tactic response (Table S1) to SAL and GABA in the presence of nonmotile cells were not significantly modified. In the presence of *M. gilvum* VM552, the net displacement and the frequency of acceleration events were slightly lower. *P. putida* G7 cell trajectories were smoother in the presence of either *M. gilvum* VM552 or *Sphingobium* sp. D4 cells with a decrease in changes of direction (Figure S2).

**Table 4:** Motility Characteristics of *Pseudomonas putida* G7, *Mycobacterium gilvum* VM552 or *Sphingobium* sp. D4 in Individual or Mixed Suspensions^a^

	*P. putida* G7			*M. gilvum* VM552		*Sphingobium* sp. D4	
	individual	+ *M. gilvum* VM552	+ *Sphingobium* sp. D4	individual	+ *P. putida* G7	individual	+ *P. putida* G7
mean instantaneous cell swimming speed (μm s^–1^)^b^	30.2 ± 9.0 (51.5)	26.3 ± 7.8 (47.1)	27.6 ± 8.6 (50.6)	8.6 ± 2.4 (14.0)	10.3 ± 2.8 (16.3)	8.7 ± 1.9 (13.2)	10.4 ± 2.3* (14.7)
net displacement in *X*-coordinate per second (μm s^–1^)^c^	31.1 ± 26.7 (117.4)	12.7 ± 11.0* (39.0)	35.7 ± 17.9 (84.6)	0.6 ± 0.2 (1.0)	3.4 ± 2.6* (11.4)	0.8 ± 0.3 (1.3)	2.8 ± 1.9* (7.5)
net displacement in *Y*-coordinate per second(μm s^–1^)^d^	23.8 ± 11.5 (45.1)	8.0 ± 5.8* (25.8)	35.6 ± 27.6 (121.9)	0.8 ± 0.3 (1.3)	3.8 ± 2.6* (10.8)	1.0 ± 0.5 (2.2)	3.6 ± 1.7* (6.7)
acceleration event frequency (no. acceleration events s^–1^)^e^	1.3 ± 1.2 (4.0)	0.3 ± 0.4* (1.7)	1.0 ± 1.3 (4.7)	1.1 ± 1.3 (4.2)	1.5 ± 1.0* (3.2)	1.3 ± 0.8 (3.1)	1.5 ± 1.8 (7.7)

a Using 1:1, carrier/cargo cell ratio. The results are presented as the mean ± the standard deviation. *, Student’s *t*-test showed significant differences compared to individual suspensions (*p* < 0.05).

b Mean instantaneous swimming speed observed for each individual cell ± standard deviation (in brackets, maximum observed value among *n* = 20).

c Position variation in *X*-coordinates per second of the total trajectory duration ± standard deviation (in brackets, maximum observed value among *n* = 20).

d Position variation in *Y*-coordinates per second of the total trajectory duration ± standard deviation (in brackets, maximum observed value among *n* = 20).

e Mean frequency of acceleration events ± standard deviation (in brackets, maximum observed value among *n* = 20).

The trajectories of *M. gilvum* VM552 and *Sphingobium* sp. D4 cells were characterized using video recordings in mixed bulk suspensions under fluorescent light (Videos S1 and S2). The instantaneous cell speed was very similar to that in the presence of *P. putida* G7, due to the Brownian movement of the cells, but the nonmotile strains traveled significantly longer distances during the recorded trajectories (Figures S3 and S4); for example, *M. gilvum* VM552 *X*- and *Y*-coordinates net displacements significantly increased by 5.7- and 4.7-fold, respectively, in the presence of *P. putida* G7. Those of *Sphingobium* sp. D4 increased by 3.5- and 3.6-fold, respectively. Furthermore, the frequency of mechanical pushes (which were determined to be acceleration events when the instantaneous speed was >20 μm s^–1^) significantly increased for *M. gilvum* VM552 in the presence of *P. putida* G7 cells but only slightly for *Sphingobium* sp. D4 (Table 4).

### Microscope Characterization of the Cotransport of *M. gilvum* VM552 and *Sphingobium* sp. D4 Nonmotile Cells with Motile *P. putida* G7 in Microarrays

An increase in the concentration of *M. gilvum* VM552 and *Sphingobium* sp. D4 cells in the microarray outlet wells were observed in the presence of *P. putida* G7, evidencing a clear enhancement of nonmotile cell dispersion within the tactic front (Table S3). Neubauer cell counting revealed that 5 times more *M. gilvum* VM552 cells and 3 times more *Sphingobium* sp. D4 cells reached the outlet well in the presence of *P. putida* G7, compared to when it was absent.

**Figure 3:**
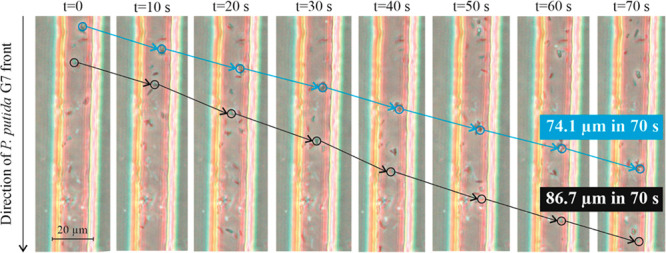
Cotransport of two example *Mycobacterium gilvum* VM552 cells in the presence of the *Pseudomonas putida* G7 chemotactic front in the capillary microarrays. The image corresponds to the left array in several photograms of the Video S3.

**Figure 4:**
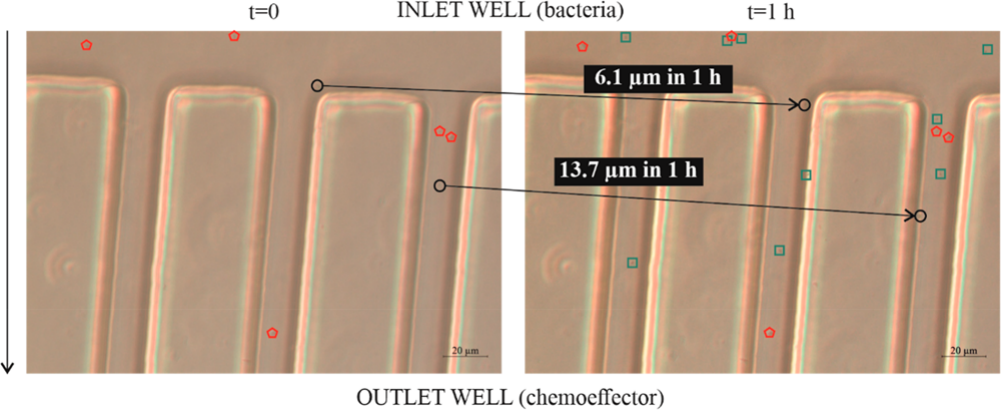
Dispersion of individual *Mycobacterium gilvum* VM552 cells in capillary microarrays. The images correspond to photographs taken with a 1 h lapse. The black circles identify cells that changed their position with time; the red pentagons denote cells that have not significantly displaced. The green squares correspond to new cells that appeared in the image after 1 h.

A detailed microscope observation of the arrays that contained mixed suspensions evidenced the mechanisms governing the cotransport of nonmotile cells within a motile tactic front in micropores. It was observed that *M. gilvum* VM552 cells were mechanically pushed and dragged by the tactic front and the turbulences created by *P. putida* G7 directional cell flow (Video S3). This result was clearly suggested by the trajectories of observed *M. gilvum* VM552 cells at different photograms (and times) in Video S3 (and represented in Figure 3). Brownian moving cells that were minimally diffusing through the narrow channels in the absence of *P. putida* G7 (Figure 4; Video S4), could actively travel ca. 80 μm after 70 s in the presence of *P. putida* G7 (or 1.1 μm s^–1^; 3 times lower than the net displacements observed in bulk suspensions, Table 4).

## Discussion

Cell-scale interactions among *P. putida* G7 and nonmotile cells of *M. gilvum* VM552 and *Sphingobium sp*. D4 were examined macroscopically in motion-limited micrometer-sized scenarios and were confirmed through examining the individual characteristics of cell motion in bulk suspensions and microarrays. Nonmotile cells could travel longer distances with *P. putida* G7 in bulk suspensions, although those displacements were 3-fold lower in microarrays, due to the impedance to cell diffusion that occurs in the narrow capillaries. In the context of our experiments, the main mechanism for the enhanced transport of nonmotile cells was the tactic motility of carrier cells. Nonmotile cells were cotransported within the *P. putida* G7 chemotactic cell front, which was directionally oriented to the chemoeffector concentration gradient that was created through pores in the devices used. This was evidenced by the bioreactor results obtained in the presence of *P. putida* G7 without chemoeffector, which were comparable to those in the absence of *P. putida* G7 (motion was only attributable to Brownian diffusion and dispersion due to a cell concentration gradient).^40^

Microscopic observations of the microarrays corroborated that nonmotile cell cotransport in the presence of the *P. putida* G7 tactic front occurred and that the main mechanisms involved mechanical pushing and hydrodynamic interactions created inside the narrow channels (mimicking pore motion regimes).^41^ This phenomenon occurred when *P. putida* G7 cells collided with nonmotile cells, which moved within the *P. putida* G7 flux in the direction of the chemoeffector concentration gradient they sensed. Cotransport also occurred as a consequence of dragging and the turbulence generated when a dense population of bacteria swim directionally inside a fluid.^42,43^ Neither multimicrobial aggregates nor the direct attachment to *P. putida* G7 cells were observed; thus, those mechanisms were excluded under our experimental conditions.

Cotransport was favored in the presence of a strong chemoeffector (i.e., GABA), which indeed generated a more significant tactic response of *P. putida* G7. This finding provided clear evidence of consolidated chemotaxis-mediated cotransport. Pore size also had a significant influence on microbe–microbe cotransport. This effect was directly related to the size of the cells, in which the length and breadth were close to 5 and 3 μm, respectively, limiting their diffusion through smaller pores, in which size exclusion occurs. An increasing pore size led to an increase in the contribution of Brownian diffusion but also enhanced the transport of chemotactic *P. putida* G7 cells and of nonmotile cells within. Previous studies on *P. putida* G7 dispersion through pores have demonstrated that larger pore sizes led to a complete homogenization of the bacterial concentration of bioreactor chambers due to a positive taxis to SAL in the upper chamber. However, this was restricted through smaller pores due to cell size restrictions and limited sensing of the chemoeffector gradient in micrometer-sized pores.^19^ Therefore, when a pore-limited taxis behavior of *P. putida* G7 existed, cotransport decreased accordingly. The average diffusion coefficients were indeed an order of magnitude higher in 12 μm bioreactors with chemoeffectors and were comparable to those of *P. putida* G7 individually. The diffusivities of passive colloids (0.5 μm-radius polystyrene spheres) in bulk suspensions were determined by Vaccari et al.^44^ and were an order of magnitude higher in the presence of motile *Pseudomonas aeruginosa* PA14Δ*pelA* (the value increased from 1.39 × 10^–7^ to 18.03 × 10^–7^ cm^2^ s^–1^). These data are consistent with our diffusivity results in 12 μm bioreactors, in which cell restriction is less limited.

In addition to the chemoeffector used and the pore size, the carrier/cargo proportion, as well as the particular surface characteristics of each bacterium, influenced cotransport. The transport of *M. gilvum* VM552 through pores was more efficient than that of *Sphingobium* sp. D4, probably due to the existence of some positive influences between the cells of *M. gilvum* VM552 and *P. putida* G7 and an enhancement of cell fitness in mixed suspensions, as well as a decreased interaction with the filters. *Sphingobium* sp. D4 is more hydrophobic and has a higher surface charge (zeta potential), which probably led to more interactions with pores and a less efficient activity by the *P. putida* G7 cells when they encounter a hydrophobic *Sphingobium* sp. D4 cell. Furthermore, the *P. putida* G7 tactic response had fewer acceleration events and predominant linear trajectories when it was coinoculated with *M. gilvum* VM552, as usually occurred in the presence of other organic chemoeffectors.^45^ This type of trajectory resulted in a smoother motility and, consequently, an enhanced transport efficiency through pores.

To our knowledge, this is the first study that demonstrates the chemotaxis-mediated microbe–microbe cotransport of HOC-degrading nonmotile cells in micrometer-sized pores that are close to cell exclusion size. Those nonmotile bacteria efficiently took advantage of the directional movement of *P. putida* G7 toward a positive chemoattractant concentration gradient, and their dispersion was significantly enhanced. Individual *M. gilvum* VM552 and *Sphingobium* sp. D4 nonmotile cells could only slightly disperse through pores due to intrinsic Brownian motion. When placed together with *P. putida* G7 in bulk suspensions, the otherwise nonmotile cells traveled further due to hydrodynamic interactions with motile cells. Cotransport was demonstrated in micropores, quantified using macroscopic observations in bioreactors and microscopically characterized in bulk suspensions and array microchannels, which allowed the mechanisms involved in microbe–microbe cotransport to be studied and demonstrated at the cell scale in micropores.

The results of this study have direct implications for the design of bioremediation inoculants for enhancing the degradation of poorly available and distant HOC fractions. Inoculants with special capabilities for HOC degradation are typically preferred, but the dispersion enhancement of those inoculants should also be considered. The combined use of motile and nonmotile strains with complementary metabolic capabilities and the application of suitable chemoeffectors will decrease the deposition rates of motile and nonmotile cells within soil particles and favor their accessibility to pores near the cell exclusion size.

## Supporting Information Available

Tactic response of *Pseudomonas putida* G7 individually of in the presence of nonmotile cells; multivariate ANOVA of cotransport of nonmotile cells; cotransport of nonmotile cells in capillary microarrays; correction factor of acridine orange fluorescence of stained nonmotile cells; detailed cell trajectories of *P. putida* G7; *Mycobacterium gilvum* VM552, and *Sphingobium* sp. D4 (PDF)
Videos S1 and S2: apparent motility of *M. gilvum* VM552 and *Sphingobium* sp. D4 in bulk suspensions in the presence of *P. putida* G7 (AVI, AVI)
Videos S3 and S4: detail of *M. gilvum* VM552 cotransport and dispersion in capillary microarrays in the presence and the absence of *P. putida* G7 (AVI, AVI)

## Supplementary Material

es2c03149_si_001.pdf

es2c03149_si_002.avi

es2c03149_si_003.avi

es2c03149_si_004.avi

es2c03149_si_005.avi
