# Supplementary material for: Chemotactic Bacteria Facilitate the Dispersion of Nonmotile Bacteria through Micrometer-Sized Pores in Engineered Porous Media
Source: Environ Sci Technol. 2022 Sep 14;56(19):13975–84. doi: 10.1021/acs.est.2c03149 (PMC9535858; doi:10.1021/acs.est.2c03149)
Supplement: Supplementary file 1 — es2c03149_si_001.pdf [file es2c03149_si_001.pdf]

SUPLEMENTARY INFORMATION

Chemotactic bacteria facilitate the dispersion of nonmotile bacteria through micrometer-sized pores in engineered porous media

*María Balseiro-Romero[1], Ángeles Prieto-Fernández[2], Leslie M. Shor[3], Subhasis Ghoshal[3], Philippe C. Baveye[5], José Julio Ortega-Calvo[1, *]*

[1] Instituto de Recursos Naturales y Agrobiología de Sevilla (IRNAS), Consejo Superior de Investigaciones Científicas (CSIC), Avda. Reina Mercedes 10, 41012 Sevilla, Spain

[2] Instituto de Investigaciones Agrobiológicas de Galicia (IIAG), Consejo Superior de Investigaciones Científicas (CSIC), Avda. de Vigo s/n, 15705 Santiago de Compostela, Spain

[3] Department of Chemical and Biomolecular Engineering, University of Connecticut, Castleman Building Rm. 224, CT 06269-3237 Storrs, United States

[4] Department of Civil Engineering, McGill University, 817 Sherbrooke Street West, H3A 0C3 Montreal, Canada

[5] Saint Loup Research Institute, 79600 Saint Loup Lamairé, France

[*]Corresponding author tel: (+34) 95-4624711; fax: (+34) 95-4624002; e-mail: jjortega@irnase.csic.es

NUMBER OF PAGES: 9

NUMBER OF TABLES: 3

NUMBER OF FIGURES: 4

NUMBER OF VIDEOS: 4

Table S1. Tactic response of *Pseudomonas putida* G7 individually or in the presence of *Sphingobium* sp. D4 or *Mycobacterium gilvum* VM552. The results are presented as the mean ± the standard deviation (*n=3*).

	individual	+ *M. gilvum* VM552	+ *Sphingobium* sp. D4
**G7 tactic factor to GABA (10 mM)**	47.5 ± 1.5	50.0 ± 0.2	52.5 ± 0.3
**G7 tactic factor to SAL (10 mM)**	6.7 ± 3.0	13.1 ± 3.7	8.5 ± 3.1

Table S2. Multivariate ANOVA analysis of C/C_0 of *Mycobacterium gilvum* VM552 and *Sphingobium* sp. D4.

	***M. gilvum* VM552**		***Sphingobium* sp. D4**	
	**F**	**Sig.**	**F**	**Sig.**
**Pore size (3, 5, 12 µm)**	9.691*	0.021	2.244*	0.015
**Chemoeffector (SAL, GABA)**	-	-	7.699*	<0.001
***P. putida* G7 cell proportion (absence, 1:1, 10:1)**	5.517*	0.009	1.501	0.136
**Pore size * Chemoeffector**	-	-	1.301	0.234
**Pore size* *P. putida* G7 cell proportion**	5.943*	0.007	1.182	0.262
**Chemoeffector * *P. putida* G7 cell proportion**	-	-	0.363	0.988

Table S3. Transported non-motile cells from inlet to outlet wells of capillary microarrays after 2 h of incubation. The results are presented as the mean percentage of cells in outlet from the total added in the inlet well ± the standard deviation (*n=2*).

	individual	G7+VM552/ D4 1:1
**% of transported *M. gilvum* VM552 nonmotile cells**	2.4 ± 3 %	11.39 ± 3.8 %
**% of transported *Sphingobium* sp. D4 nonmotile cells**	1.8 ± 0.2%	5.6 ± 2.5%

Figure S1. Determination of the CF of A) *Mycobacterium gilvum* VM552 and B) *Sphingobium* sp. D4 for AO fluorescence correction according to the total optical density of the suspension (Eq. 1 and 2, respectively).

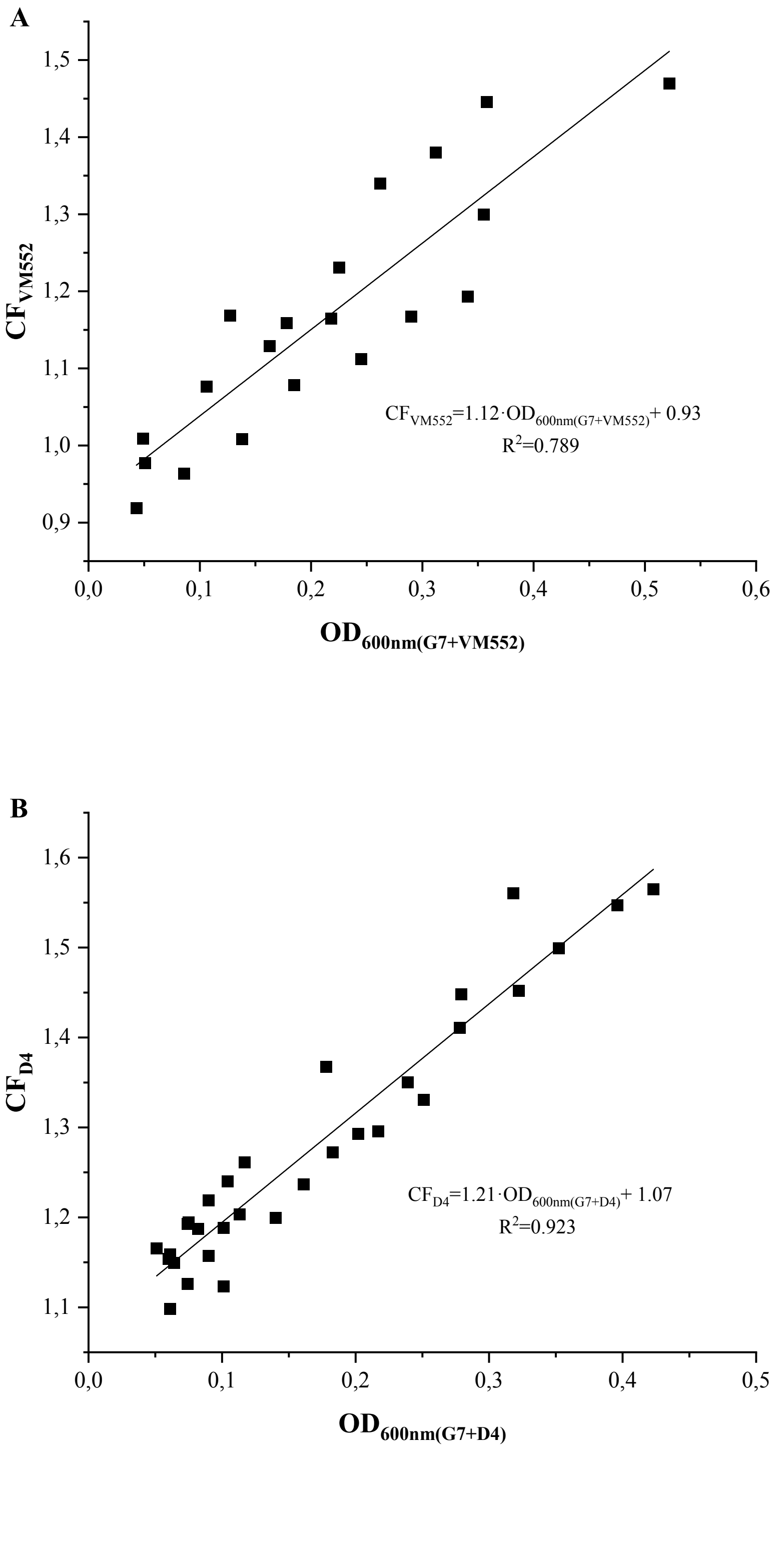

Figure S2. Coordinates of the cell trajectories of *Pseudomonas putida* G7 in the videos recorded from bulk suspensions A) individually; B) in the presence of *Mycobacterium gilvum* VM552; and C) in the presence of *Sphingobium* sp. D4. The videos were recorded for 6-7 seconds.

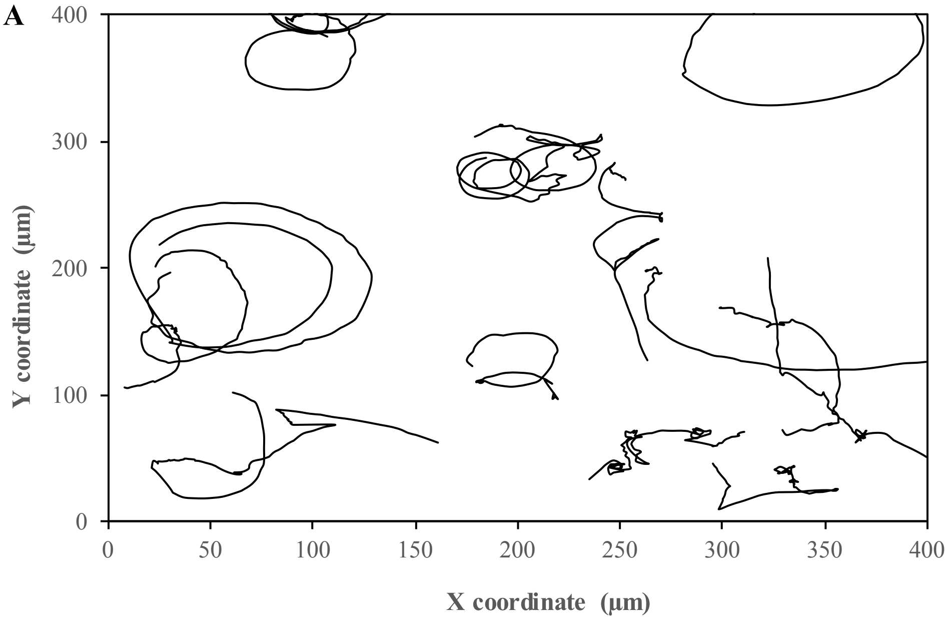

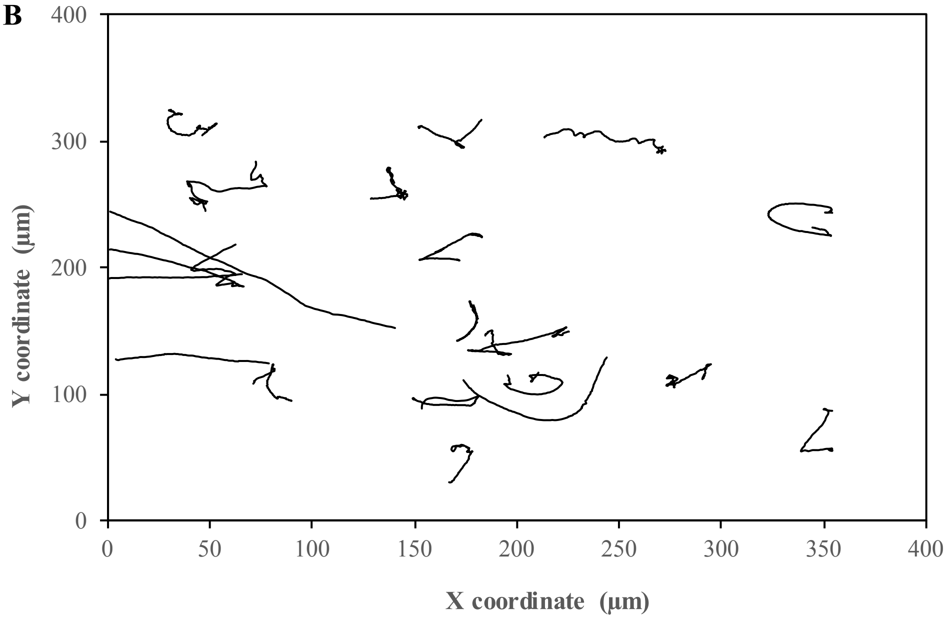

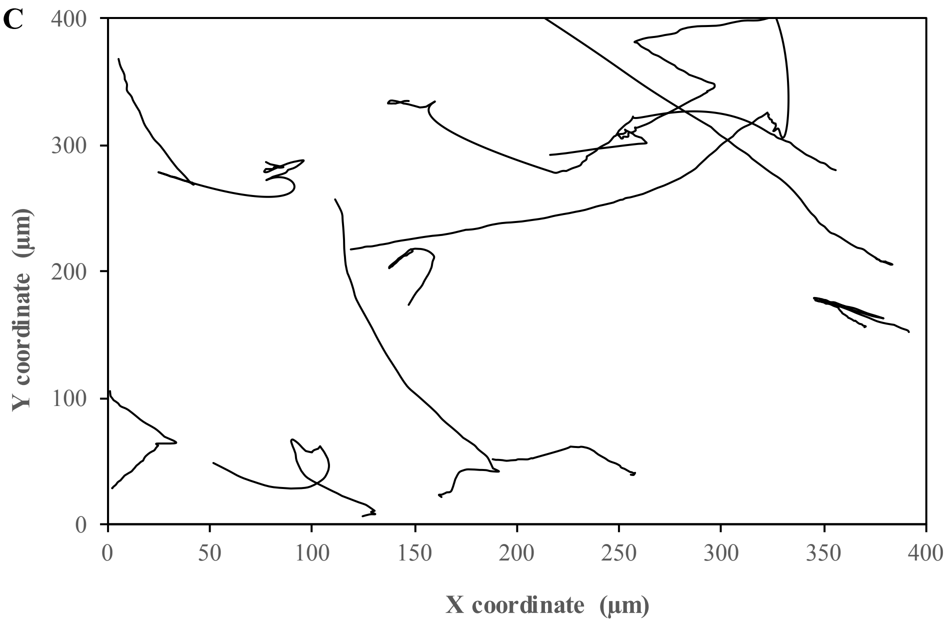
C
400
300
200
100
0
Y coordinate (µm)
0
50
100
150
200
250
300
350
400
X coordinate (µm)

Figure S3. Coordinates of *Mycobacterium gilvum* VM552 cell trajectories in the videos recorded from bulk suspensions A) individually; and B) in the presence of *Pseudomonas putida* G7. The videos were recorded for 13 seconds.

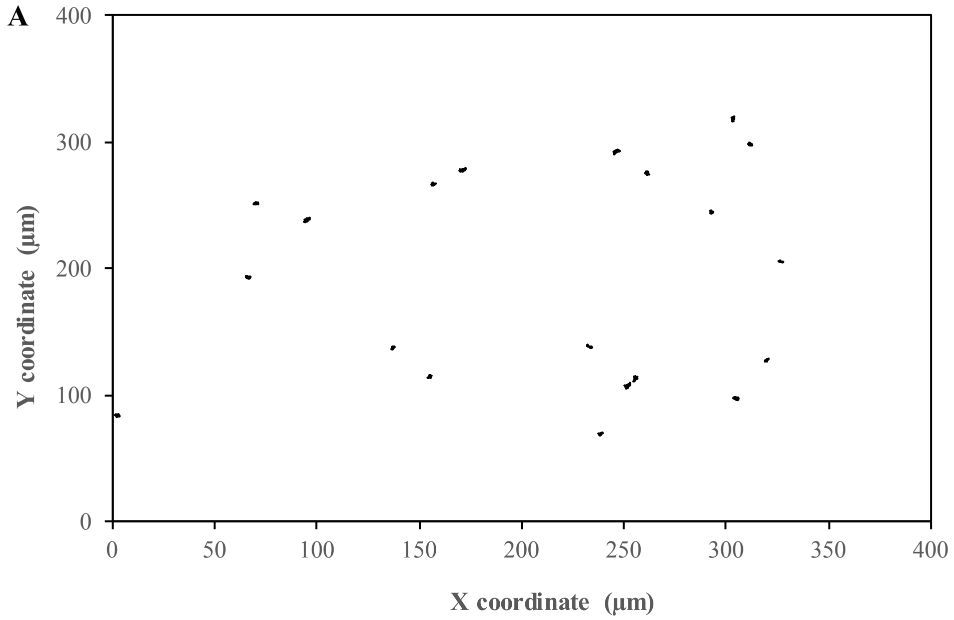

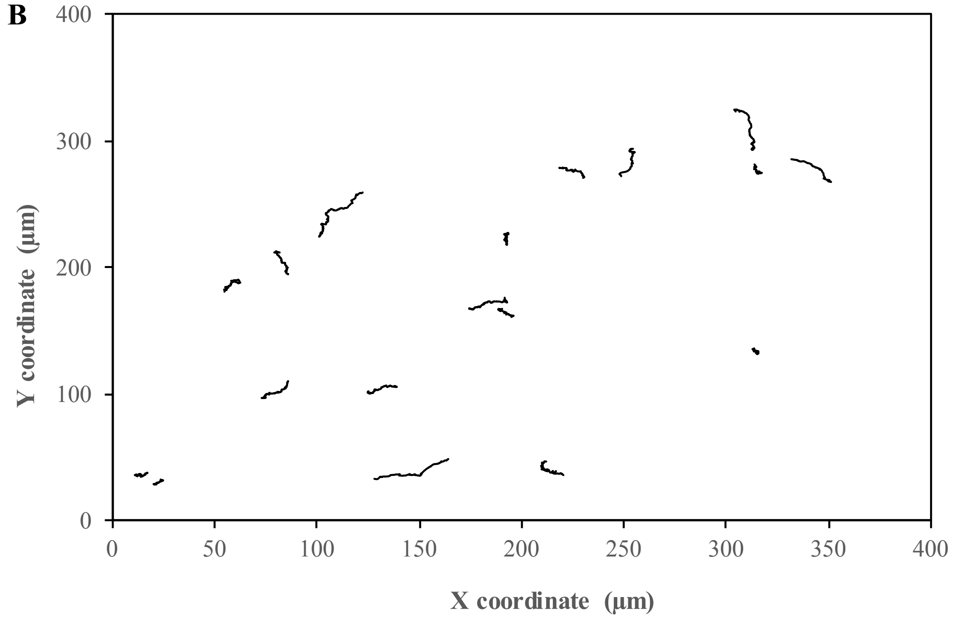

Figure S4. Coordinates of *Sphingobium* sp. D4 cell trajectories in the videos recorded from bulk suspensions A) individually; and B) in the presence of *Pseudomonas putida* G7 (Video S2). The videos were recorded for 8 seconds.

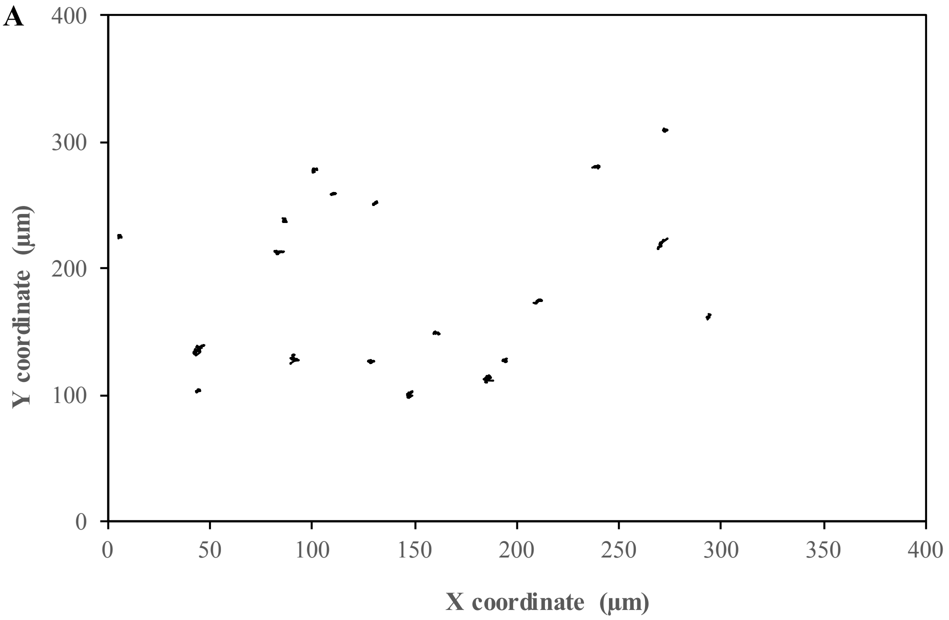

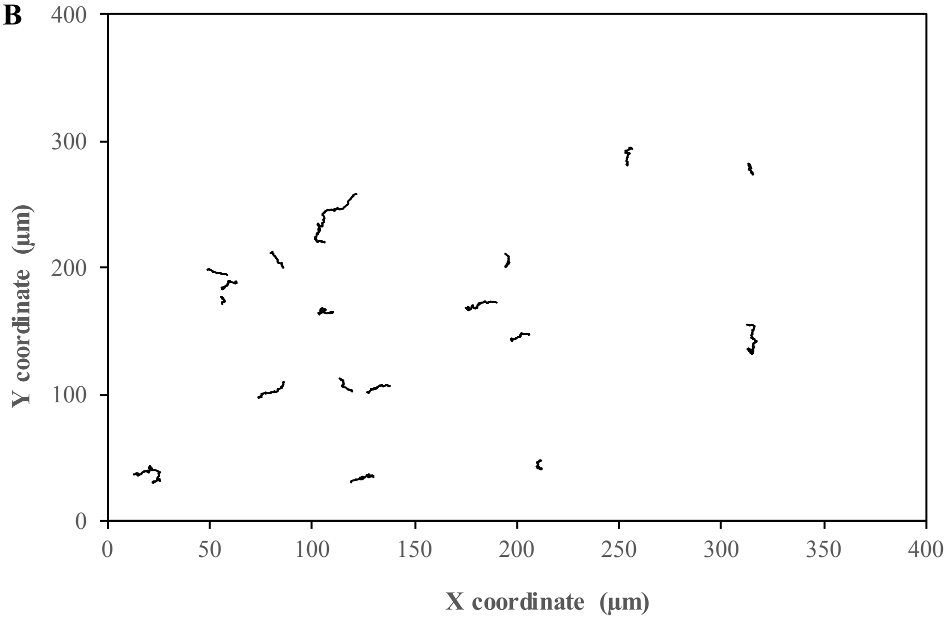

VIDEOS

Video S1. Video of *Mycobacterium gilvum* VM552 apparent motility under fluorescent light, in the presence of motile *Pseudomonas putida* G7 in bulk suspensions.
https://saco.csic.es/index.php/s/6ajjQaHAw6GzKFP

Video S2. Video of *Sphingobium* sp. D4 apparent motility under fluorescent light, in the presence of motile *Pseudomonas putida* G7 in bulk suspensions.
https://saco.csic.es/index.php/s/FckSNPtp48Yo2gq

Video S3. Detail of *Mycobacterium gilvum* VM552 cotransport along the microarray channels in the presence of motile *Pseudomonas putida* G7 cells.
https://saco.csic.es/index.php/s/gJGESM2YxaKR67e

Video S4. Detail of *Mycobacterium gilvum* VM552 dispersion along the microarray channels in the absence of *Pseudomonas putida* G7 cells. The video was recorded from the moment the right picture of Figure S3 was taken. The Brownian movement of cells can be easily observed.
https://saco.csic.es/index.php/s/ZW2Nc8w9YkjDTpb
